# Association Between TyG‐BMI Index and Hyperuricemia in Adult Women

**DOI:** 10.1002/edm2.70028

**Published:** 2025-02-03

**Authors:** Kelibinuer Mutailipu, Junwei Guo, Jiajing Yin, Yue Wang, Liesheng Lu, Xuyang Jia, Jie Zhang, Shen Qu, Haibing Chen, Le Bu

**Affiliations:** ^1^ Department of Endocrinology and Metabolism, Shanghai Tenth People's Hospital Institute of Obesity, School of Medicine, Tongji University Shanghai People's Republic of China; ^2^ Department of Metabolic Surgery, Shanghai Tenth People's Hospital Institute of Obesity, School of Medicine, Tongji University Shanghai People's Republic of China; ^3^ Department of Clinical Laboratory Medicine, Shanghai Tenth People's Hospital, School of Medicine Tongji University Shanghai People's Republic of China

**Keywords:** BMI, hyperuricemia, TyG index, TyG‐BMI index, women

## Abstract

**Purpose:**

This study aimed to explore the relationship between hyperuricemia (HUA), the triglyceride‐glucose index (TyG) and its derivatives in adult women.

**Methods:**

A cross‐sectional analysis was conducted on 1105 female patients from Shanghai Tenth People's Hospital. Participants were divided into HUA (*n* = 331) and non‐HUA (*n* = 774) groups. Clinical and laboratory data were collected, and indices such as body mass index (BMI), TyG and TyG‐BMI were calculated. Statistical analyses included univariate and multivariate logistic regression and receiver operating characteristic (ROC) curve analysis.

**Results:**

The HUA group showed higher BMI, blood pressure and metabolic parameters. TyG, TyG‐BMI and BMI were positively correlated with uric acid levels. ROC analysis revealed that TyG‐BMI (AUC = 0.877) had better predictive power for HUA than TyG (AUC = 0.829) or BMI (AUC = 0.867). Multivariate analysis showed TyG‐BMI and BMI as independent predictors, with women in the highest quartiles having a 3.111‐fold and 2.779‐fold higher risk for HUA, respectively.

**Conclusion:**

TyG‐BMI is the most effective predictor of HUA in women, surpassing TyG and BMI alone. It offers a practical tool for early identification and intervention in women at risk of HUA.

## Graphical abstract

TyG‐BMI demonstrates the strongest predictive power, emphasizing its utility as a practical tool for identifying hyperuricemia (HUA) risk in women.

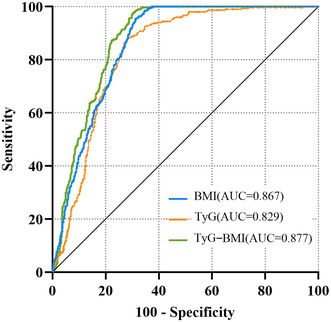

## 1 Introduction

Uric acid (UA) is the final product of purine nucleotide metabolism, with 80% derived from the breakdown of nuclear proteins and 20% from purine‐rich dietary sources [1, 2]. Hyperuricemia (HUA) is typically attributed to impaired uric acid excretion, excessive production or both [3]. Notably, the prevalence of HUA has significantly increased over the past two decades, with reports indicating a prevalence of 17.4% in mainland China, including 11.0% in women, highlighting sex differences in HUA risk [4, 5]. Although the overall prevalence of HUA is lower in women than in men, it remains a significant risk factor for cardiovascular and metabolic disorders. Elevated serum uric acid (SUA) levels have been shown to be associated with arterial stiffness, ischemic stroke, coronary heart disease (CHD) and non‐alcoholic fatty liver disease (NAFLD) in women [6, 7, 8, 9, 10], as evidenced by studies showing a heightened risk for these conditions in female populations. These findings underscore the broad range of health issues related to HUA in women, emphasising its clinical significance.

Epidemiological studies have consistently shown strong correlations between elevated SUA and insulin resistance (IR) [11]. The relationship between IR and HUA is bidirectional, with hyperinsulinemia reducing uric acid excretion and promoting its production, exacerbating both conditions [12, 13]. Given IR's role in HUA development, IR assessment is essential for identifying at‐risk individuals. While the hyperinsulinemic‐euglycemic clamp (HIEC) is the gold standard for detecting IR, its complexity limits clinical use [14]. In contrast, the triglyceride‐glucose (TyG) index, a simpler and reliable alternative, correlates strongly with IR in both diabetic and non‐diabetic populations [15, 16], and indices like TyG‐BMI may further improve early detection of IR [17].

The TyG index and its derivatives, such as TyG‐BMI, are strong predictors of insulin resistance (IR) and elevated serum uric acid (SUA) levels, particularly in individuals with type 2 diabetes and metabolic disorders [18, 19]. When combined with obesity‐related metrics, such as waist‐to‐height ratio (WHtR), the TyG index further enhances its predictive power for hyperuricemia, especially in women [20]. Although men typically have a higher prevalence of hyperuricemia, women are more vulnerable to the cardiovascular and metabolic complications associated with the condition. However, research specifically examining the relationship between the TyG index and hyperuricemia in adult women remains limited. This study aims to address this gap and test the hypothesis that the TyG index and its derivatives can effectively predict hyperuricemia in this high‐risk population.

## 2 Methods

### 2.1 Research Population

**FIGURE 1:**
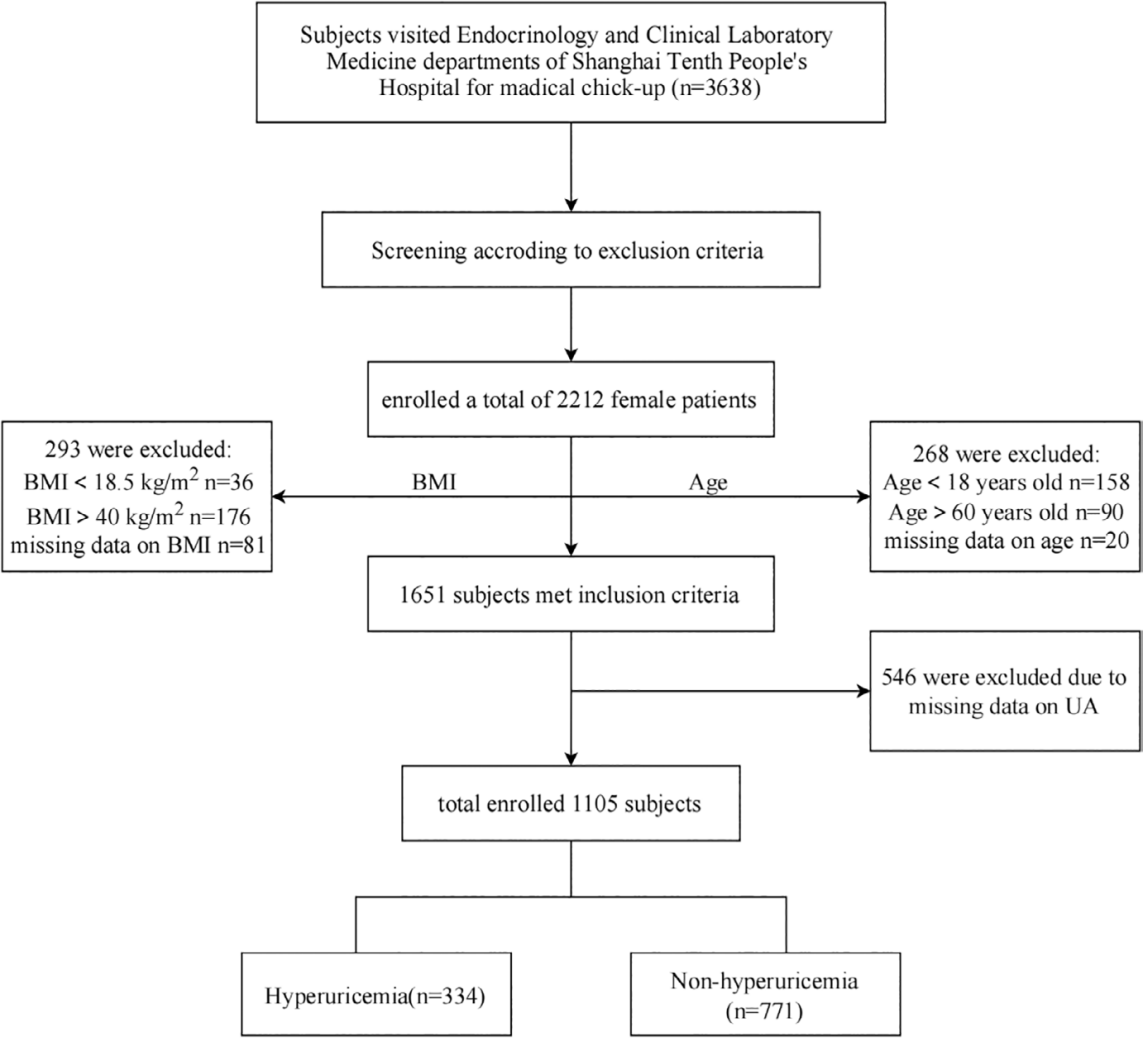
Study flow chart.

This cross‐sectional study retrospectively analysed 1105 adult female patients treated at the Endocrinology and Clinical Laboratory Medicine departments of Shanghai Tenth People's Hospital between September 2009 and September 2021. Figure 1 presents a flowchart of the study. HUA was defined as an SUA level of > 357 μmol/L in females, with no clinical symptoms associated with hyperuricemia. The subjects were divided into HUA (*n* = 331) and non‐HUA (*n* = 774) groups. The study was approved by the Ethics Committee of Shang Hai Tenth People's Hospital (approval no. 2012‐RES‐05) and was conducted in accordance with the Declaration of Helsinki. The requirement for informed consent was waived owing to the retrospective nature of the analysis.

### 2.2 Clinical and Laboratory Data

Demographic data, including sex, age, heart rate (HR), systolic blood pressure (SBP), diastolic blood pressure (DBP), weight and height, were obtained from electronic medical records. Following an 8‐h fasting period, weight and height were measured using standardised equipment with participants in lightweight clothing and without shoes. Venous blood samples were then collected and analysed for UA, FG, total cholesterol (TC), TG, high‐density lipoprotein (HDL), low‐density lipoprotein (LDL), alanine aminotransferase (ALT), serum creatinine (Scr) and blood urea nitrogen (BUN). The BMI, TyG index and TyG‐BMI index were calculated using the following equations:

$$\mathrm{BMI}=\text{weight}\;\left(\mathrm{kg}\right)/\left[\text{height}\;\left(m\right)\right].$$

$$\mathrm{TyG}=\ln\;\left[\mathrm{TG}\;\left(\mathrm{mg}/\mathrm{dL}\right)\times \mathrm{FPG}\;\left(\mathrm{mg}/\mathrm{dL}\right)/2\right].$$

TyG−BMI=TyG×BMI.

### 2.3 Statistical Analysis

SPSS version 26.0; GraphPad Prism 8.0, and R version 4.3.1. was used for the statistical analyses. The Shapiro–Wilk test was used to assess the normality of continuous variables. Normally distributed data are presented as mean ± SD and analysed using the independent *t*‐test, whereas non‐normally distributed data are presented as median (IQR) and compared using the Mann–Whitney *U* test. Categorical variables were compared using chi‐squared or Fisher's exact tests. Univariate linear regression was used to assess the association between clinical factors and serum uric acid levels. Univariate logistic regression was used to identify the predictors of hyperuricemia. Receiver operating characteristic (ROC) curves were used to calculate the area under the curve (AUC), sensitivity, specificity and cutoff points for BMI, TyG and TyG‐BMI. Multivariate logistic regression models were used to examine independent associations with hyperuricemia. Statistical significance was set at *p* < 0.05.

## 3 Results

### 3.1 Baseline Characteristics of the Participants

The clinical characteristics of the patients are summarised in Table 1. The HUA group, despite a similar average age to the non‐HUA group, exhibited significantly higher BMI (33.6 kg/m^2^ (5.8) vs. 22.4 kg/m^2^ (9.5), *p* < 0.001) and elevated SP and diastolic BP. Metabolically, patients with HUA showed higher FPG, TC and TG levels, whereas LDL levels were significantly higher, and HDL levels were significantly lower. Liver function tests indicated elevated ALT and Cr levels but no significant difference in BUN levels.

**TABLE 1:** Clinical characteristics of participants by the presence of HUA.

Variables	HUA (*N* = 331)	Non‐HUA (*N* = 774)	*p*
UA (umol/L)	417.8 (71.6)	282.0 (64.4)	< 0.001
Age (years)	30.0 (10.0)	30.0 (11.0)	0.853
HR (beats/min)	84.0 (17.0)	82.0 (15.0)	< 0.001
SBP (mmHg)	130.0 (20.0)	117.5 (19.0)	< 0.001
DBP (mmHg)	83.0 (15.0)	72.0 (15.3)	< 0.001
Weight (Kg)	89.8 ± 12.1	83.3 ± 12.5	< 0.001
Height (m)	1.6 ± 0.9	1.6 ± 0.8	0.007
BMI (kg/m^2^)	33.6 (5.8)	22.4 (9.5)	< 0.001
FPG (mmol/L)	5.4 (1.2)	4.5 (0.8)	< 0.001
TC (mmol/L)	4.9 (1.3)	4.4 (1.0)	< 0.001
TG (mmol/L)	1.7 (1.0)	0.9 (0.6)	< 0.001
HDL (mmol/L)	1.1 (0.3)	1.3 (0.3)	< 0.001
LDL (mmol/L)	3.9 (1.2)	2.5 (0.9)	< 0.001
ALT (U/L)	39.0 (44.6)	14.0 (11.0)	< 0.001
Cr (umol/L)	58.8 (12.3)	56.0 (10.0)	0.006
BUN (mmol/L)	4.4 (1.1)	4.20 (1.5)	0.113
TyG	8.9 (0.7)	8.0 (0.8)	< 0.001
TyG‐BMI	304.9 (53.5)	175.3 (93.4)	< 0.001

Abbreviations: ALT, alanine aminotransferase; BMI, body mass index; BUN, blood urea nitrogen; Cr, creatinine; DBP, diastolic blood pressure; FPG, fasting plasma glucose; HDL, high‐density lipoprotein cholesterol; HR, heart rate; LDL, low‐density lipoprotein cholesterol; SBP, systolic blood pressure; TC, total cholesterol; TG, triglycerides; TyG, triglyceride–glucose index; TyG‐BMI, triglyceride–glucose index body mass index.; UA, uric acid.

### 3.2 Associations Between Serum Uric Acid and Key Clinical Indices

**FIGURE 2:**
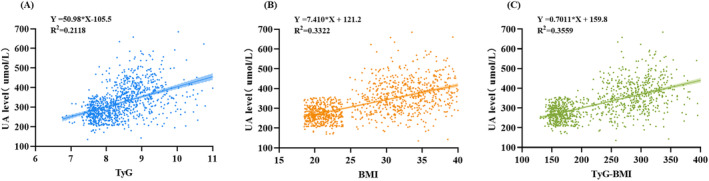
The correlation between TyG index and UA (A), BMI and UA (B) and TyG‐BMI and UA (C).

Table S1 presents the results of univariate linear regression analyses examining the relationship between UA levels and various clinical and laboratory parameters. Significant positive correlations were observed between UA levels and BMI, HR, SP, DP, FPG, TC, TG, HDL, LDL, ALT, Cr, TyG and TyG BMI (*p* < 0.05). Among these, the TyG index accounted for 21.2% of the variance in SUA levels (*R* ^2^ = 0.212; Figure 2A), reflecting a notable positive correlation. BMI showed a stronger association with SUA level, explaining 33.2% of the variance (*R* ^2^ = 0.332; Figure 2B). TyG‐BMI demonstrated the highest explanatory power, accounting for 35.6% of the variance in SUA levels (*R* ^2^ = 0.356; Figure 2C).

Predictive Value of BMI, TyG and TyG‐BMI for Hyperuricemia.

**FIGURE 3:**
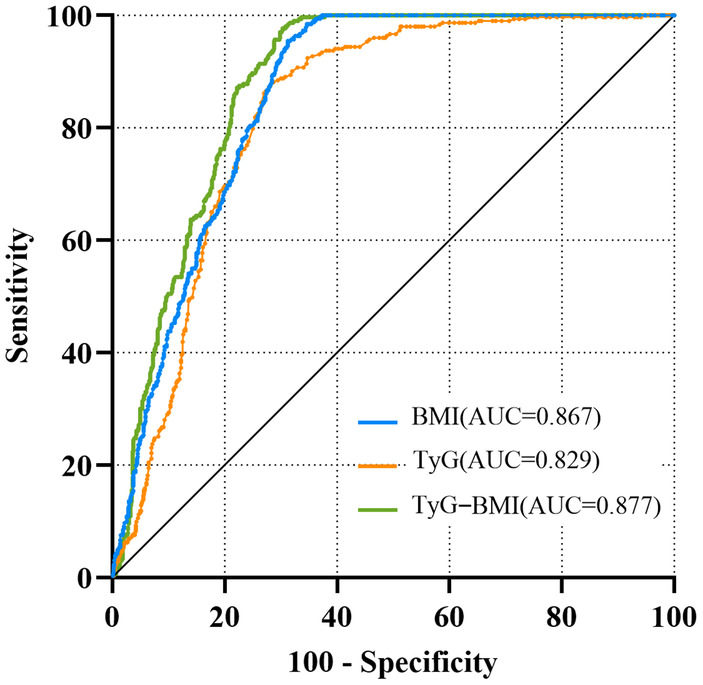
ROC curve of TyG index, BMI and TyG‐BMI in predicting HUA in adult female patients.

**FIGURE 4:**
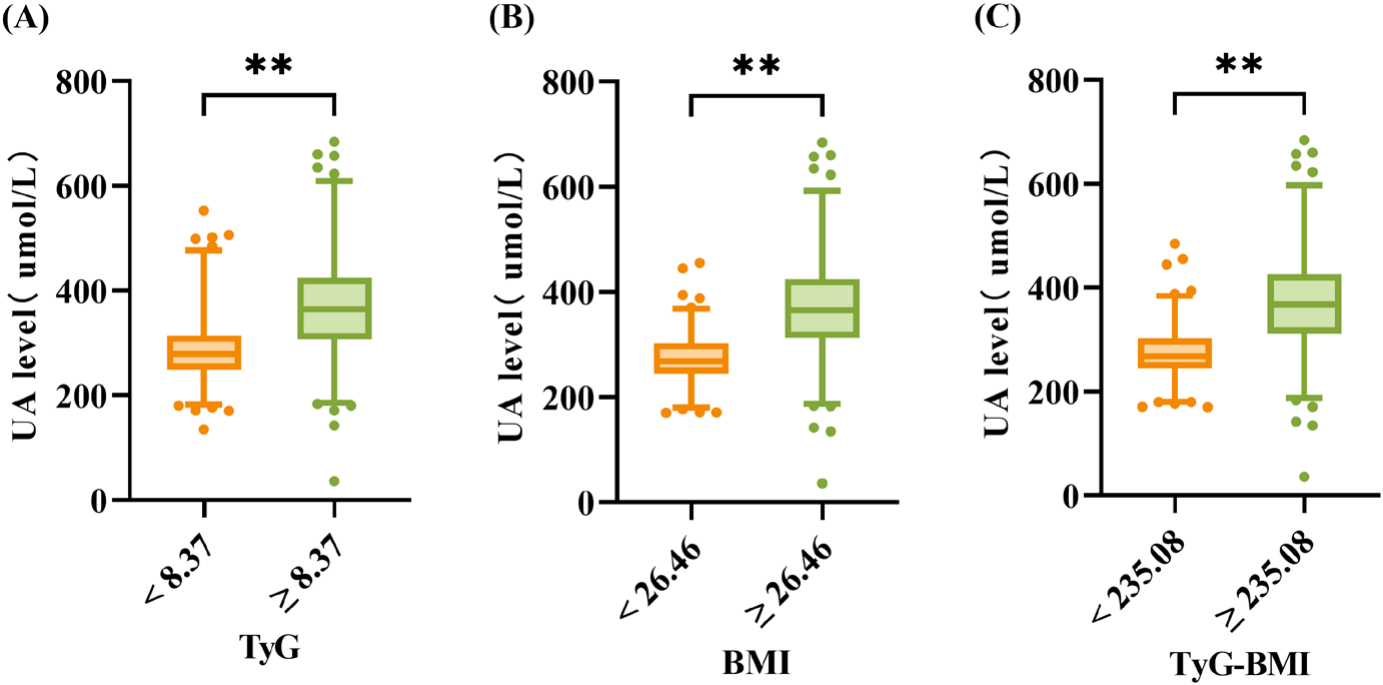
Box plots of TyG, BMI, TyG‐BMI and UA. ** indicates a highly significant difference between the two groups, with a *p*‐value less than 0.001.

Table S2 presents the univariate logistic regression analysis of HUA, identifying several significant predictors including BMI, TyG and TyG‐BMI (*p* < 0.05). These three indices were further assessed for their predictive value using the ROC curve analysis, as shown in Figure 3 and Table 2. TyG‐BMI demonstrated the highest area under the curve (AUC = 0.877), followed by BMI (AUC = 0.867), and TyG (AUC = 0.829). Regarding sensitivity and specificity, TyG‐BMI achieved a sensitivity of 97.7% and a specificity of 69.7%, confirming it as the strongest predictor of hyperuricemia. Additionally, Figure 4 illustrates that individuals with values above the optimal thresholds for each index (235.1 for TyG‐BMI, 26.5 for BMI and 8.4 for TyG) consistently had higher median serum uric acid levels.

**TABLE 2:** Sensitivity, specificity, positive likelihood ratio, negative likelihood ratio, and Youden index comparing various thresholds of BMI, TyG index and TyG‐BMI in female patients with hyperuricemia.

Variables	AUC	Cut‐off	Sensitivity (%)	Specificity (%)	+LR	−LR	Youden index
BMI	0.867	26.5	98.4 (96.2–99.5)	67.2 (63.7–70.5)	2.99 (2.65–3.37)	0.03 (0.01–0.06)	0.66
TyG	0.829	8.4	88.1 (83.9–91.5)	71.3 (67.9–74.6)	3.07 (2.61–3.60)	0.17 (0.11–0.24)	0.59
TyG‐BMI	0.877	235.1	97.7 (95.3–99.1)	69.7 (66.3–73.0)	3.23 (2.83–3.67)	0.03 (0.01–0.07)	0.67

*Note:* Values in parentheses are 95% confidence intervals.

Abbreviations: +LR, positive likelihood ratio; −LR, negative likelihood ratio.

### 3.3 Multifactor Logistic Regression Analysis

In Table 3, multifactorial logistic regression analysis presents the associations between BMI, TyG, TyG‐BMI and HUA. The results were based on the optimal cut‐off points identified through ROC curve analysis. In the unadjusted model, BMI (OR = 1.11, 95% CI = 1.06–1.16, *p* < 0.001) and TyG‐BMI*0.1 (OR = 1.10, 95% CI = 1.05–1.16, *p* < 0.001) were independently associated with HUA. After adjusting for covariates, the BMI and TyG‐BMI remained significantly correlated. Women in the highest BMI quartile had a 2.78‐fold higher risk of HUA (AOR = 2.78, 95% CI = 1.18–6.53, *p* = 0.019), while those in the highest TyG‐BMI quartile had a 3.11‐fold higher risk (AOR = 3.11, 95% CI = 1.22–7.93, *p* = 0.017), both of which showed significant trends. However, TyG alone did not demonstrate a significant independent association in any of the models (AOR = 1.18, 95% CI = 0.54–2.61, *p* = 0.677).

**TABLE 3:** Multivariate logistic regression analysis of the associations between BMI, TyG, TyG‐BMI, and hyperuricemia.

	HUA number (%)	Model 1		Model 2		Model 3	
		AOR (95%CI)	*p*	AOR (95%CI)	*p*	AOR (95%CI)	*p*
BMI (median [range])	326 (54.7)	1.11 (1.06–1.16)	< 0.001	1.11 (1.05–1.17)	< 0.001	1.11 (1.02–1.20)	0.016
Q1 (28.58 [≤ 30.12])	64 (43.0)	1 (Reference)	< 0.001	1 (Reference)	0.001	1 (Reference)	0.112
Q2 (31.51 [30.12–33.02])	79 (53.0)	1.50 (0.95–2.37)	0.083	1.49 (0.91–2.44)	0.109	1.67 (0.69–4.02)	0.253
Q3 (34.47 [33.02–35.67])	82 (55.4)	1.65 (1.04–2.61)	0.032	1.65 (1.01–2.70)	0.045	2.17 (0.92–5.08)	0.075
Q4 (37.37 [> 35.67])	101 (67.3)	2.74 (1.71–4.38)	< 0.001	2.87 (1.72–4.81)	< 0.001	2.78 (1.18–6.53)	0.019
P trend		< 0.001		< 0.001		0.016	
TyG*10 (median [range])	268 (55.1)	1.00 (0.97–1.04)	0.802	1.00 (0.96–1.003)	0.824	1.02 (0.94–1.10)	0.677
Q1 (8.51 [≤ 8.62])	56 (45.9)	1 (Reference)	0.111	1 (Reference)	0.352	1 (Reference)	0.660
Q2 (8.76 [8.62–8.89])	69 (57.0)	1.56 (0.94–2.60)	0.083	1.28 (0.74–2.20)	0.378	1.06 (0.49–2.26)	0.886
Q3 (9.11 [8.89–9.31])	74 (60.7)	1.82 (1.09–3.02)	0.021	1.66 (0.96–2.86)	0.072	1.59 (0.72–3.49)	0.250
Q4 (9.66 [> 9.31])	69 (57.0)	1.56 (0.94–2.60)	0.083	1.23 (0.71–2.12)	0.455	1.11 (0.42–2.94)	0.833
P trend		0.127		0.459		0.637	
TyG‐BMI*0.1 (median [range])	296 (56.7)	1.10 (1.05–1.16)	< 0.001	1.10 (1.04–1.16)	0.001	1.10 (1.00–1.20)	0.044
Q1 (252.17 [≤ 268.83])	57 (43.5)	1 (Reference)	< 0.001	1 (Reference)	0.002	1 (Reference)	0.031
Q2 (284.20 [268.83–295.79])	72 (55.4)	1.61 (0.99–2.63)	0.056	1.47 (0.87–2.48)	0.148	1.22 (0.51–2.89)	0.660
Q3 (308.35 [295.79–320.29])	76 (58.0)	1.79 (1.10–2.93)	0.019	1.720 (1.01–2.91)	0.044	1.13 (0.48–2.69)	0.780
Q4 (337.75 [> 320.29])	91 (70.0)	3.03 (1.82–5.04)	< 0.001	3.03 (1.73–5.32)	< 0.001	3.11 (1.22–7.93)	0.017
P trend		< 0.001		< 0.001		0.015	

*Note:* Model1: unadjusted. Model2: adjusted for HR, SBP, DBP. Model3: further adjusted for FPG, TC, HDL, LDL, ALT and Cr.

## 4 Discussion

This study identified TyG‐BMI as the strongest independent predictor of HUA in adult women, outperforming both BMI and the TyG index. In the ROC analysis, TyG‐BMI had the highest area under the curve AUC (0.877) with a sensitivity of 97.69% and specificity of 69.72%. Women with TyG‐BMI values exceeding 235.08 exhibited significantly higher median serum uric acid levels, even after adjusting for multiple confounders. Although BMI was also significantly associated with HUA, the TyG index alone was not an independent predictor in the general population.

Several previous studies, including those by Liu et al. [21] and Zhou et al. [22], have reported a significant association between the TyG index and HUA in specific populations, including hypertensive patients and female university students. However, our study did not find TyG to be an independent risk factor of HUA in adult women. This discrepancy could be attributed to differences in study populations and age, as well as hormonal factors, particularly oestrogen, which promotes uric acid excretion by modulating intestinal uric acid transport proteins such as ABCG2 through the PI3K/Akt signalling pathway [23, 24] and is associated with reduced uric acid reabsorption in the renal tubules [25, 26]. Uric acid levels in women also fluctuate during the menstrual cycle and peak during the follicular phase [27]. A Chinese study on peri‐menopausal women found that post‐menopausal declines in estradiol increased the HU risk [28]. In our study, with a median participant age of 30 years, higher oestrogen levels likely provided some protective effects, reducing the impact of TyG index on uric acid levels.

In contrast to the TyG index, BMI consistently emerged as a significant independent risk factor for HUA, consistent with the findings of Piao et al. [29] and He et al. [30]. An elevated BMI is associated with higher uric acid production and reduced excretion. Adipose tissue creates a hypoxic environment that upregulates xanthine oxidoreductase (XOR) activity, thereby increasing uric acid production from purines [31, 32]. Additionally, pentose phosphate pathway activation during fatty acid synthesis in adipose tissue contributes to increased uric acid production [33, 34]. Insulin resistance in obesity elevates adenosine levels, causing sodium retention and decreased uric acid excretion [35]. These mechanisms account for our finding that women in the highest BMI quartile had a 2.779‐fold higher risk of HUA even after adjusting for multiple confounders.

While BMI alone is a strong predictor of HUA, TyG‐BMI's superior predictive ability lies in its capacity to integrate insulin resistance and obesity, which are two key factors that independently influence uric acid metabolism. For instance, insulin resistance enhances renal reabsorption of uric acid by increasing the activity of transporters, such as GLUT9 and URAT1, while simultaneously inhibiting ABCG2‐mediated uric acid excretion [36, 37, 38]. Obesity exacerbates this situation by promoting adipose tissue accumulation and further increasing uric acid production [39]. These two mechanisms interact to create a vicious cycle wherein elevated uric acid levels further worsen insulin resistance [40, 41]. TyG‐BMI effectively captured the combined effect of both metabolic abnormalities, explaining its exceptional performance as a predictor of HUA.

Despite these compelling findings, our study had several limitations. First, we did not account for menstrual cycle phases, which could introduce variability in uric acid levels owing to hormonal fluctuations, particularly oestrogen's role in modulating uric acid metabolism. Second, key socioeconomic and lifestyle factors, such as education, smoking, physical activity and dietary factors (e.g., alcohol and meat consumption), were not considered, which could influence uric acid levels and introduce bias. Third, medication use was not systematically collected, although certain medications may affect TyG and uric acid levels. Finally, the cross‐sectional design limits our ability to establish causal relationships between TyG‐BMI, BMI and HUA. Although no formal power analysis was conducted, future longitudinal studies could benefit from such an analysis to validate and further clarify the relationship between TyG‐BMI and HUA across different stages of a woman's life.

## Author Contributions

All authors met the authorship requirements. Kelibinuer Mutailipu and Junwei Guo contributed equally to this work as co‐first authors. Le Bu conceived of and designed the study. Data collection was performed by Kelibinuer Mutailipu, Junwei Guo, JiajingYin, Yue Wang, Jie Zhang and Liesheng Lu. Haibing Chen was involved in study design, data collection and analysis. Xuyang Jia and Shen Qu performed the statistical analyses. The first draft of the manuscript was written by Kelibinuer Mutailipu and Junwei Guo, and all the authors have provided critical revisions. Le Bu supervised the project. All authors reviewed and approved the final version of the manuscript.

## Ethics Statement

The study was approved by the Research Ethics Review Committee of Shanghai 10th People's Hospital (approval no. 2012‐RES‐05). This study was conducted in accordance with the principles of the Declaration of Helsinki and updated in 2013. Owing to the retrospective design of this study, the requirement for informed consent was waived by the Ethics Committee.

## Consent

All authors have provided their consent for publication.

## Conflicts of Interest

The authors declare no conflicts of interest.

## Supporting information


**Table S1.** Univariate Linear Regression Analysis of Serum Uric Acid Levels.

**Table S2.** Univariate Logistic Regression Analysis of Hyperuricemia (HUA).

## Data Availability

The datasets generated and analysed during the current study are not publicly available due to ethical approval conditions but are available from the corresponding author upon reasonable request.
