# Supplementary material for: Association Between TyG‐BMI Index and Hyperuricemia in Adult Women
Source: Endocrinol Diabetes Metab. 2025 Feb 3;8(2):e70028. doi: 10.1002/edm2.70028 (PMC11791015; doi:10.1002/edm2.70028)
Supplement: Supplementary file 1 — Table S1. Univariate Linear Regression Analysis of Serum Uric Acid Levels. [file EDM2-8-e70028-s002.docx]

Supplementary Table 1: Univariate Linear Regression of Serum Uric Acid

|  | B | t | R | R^2^ | P |
| --- | --- | --- | --- | --- | --- |
| BMI | 7.409 | 23.425 | 0.576 | 0.332 | 0.000 |
| HR | 0.957 | 4.495 | 0.139 | 0.019 | 0.000 |
| SBP | 1.356 | 9.735 | 0.287 | 0.082 | 0.000 |
| DBP | 2.196 | 12.355 | 0.356 | 0.127 | 0.000 |
| FPG | 9.168 | 6.849 | 0.205 | 0.042 | 0.000 |
| TC | 18.417 | 6.956 | 0.208 | 0.043 | 0.000 |
| TG | 26.576 | 12.139 | 0.348 | 0.121 | 0.000 |
| HDL | -61.900 | -7.300 | 0.219 | 0.048 | 0.000 |
| LDL | 24.589 | 7.802 | 0.234 | 0.055 | 0.000 |
| ALT | 1.004 | 14.580 | 0.408 | 0.167 | 0.000 |
| Cr | 1.187 | 4.467 | 0.158 | 0.025 | 0.000 |
| TyG | 50.995 | 16.754 | 0.460 | 0.212 | 0.000 |
| TyG-BMI | 0.701 | 24.021 | 0.597 | 0.356 | 0.000 |
