# Supplementary material for: Association Between TyG‐BMI Index and Hyperuricemia in Adult Women
Source: Endocrinol Diabetes Metab. 2025 Feb 3;8(2):e70028. doi: 10.1002/edm2.70028 (PMC11791015; doi:10.1002/edm2.70028)
Supplement: Supplementary file 2 — Table S2. Univariate Logistic Regression Analysis of Hyperuricemia (HUA). [file EDM2-8-e70028-s001.docx]

| Variables | OR(95%CI) | P value |
| --- | --- | --- |
| BMI | 1.301（1.260-1.343） | ＜0.001 |
| HR | 1.024（1.013-1.036） | ＜0.001 |
| SBP | 1.040（1.031-1.049） | ＜0.001 |
| DBP | 1.069（1.056-1.081） | ＜0.001 |
| FPG | 1.330（1.220-1.449） | ＜0.001 |
| TC | 1.871（1.601-2.187） | ＜0.001 |
| TG | 2.297（1.928-2.737） | ＜0.001 |
| HDL | 0.136（0.077-0.241） | ＜0.001 |
| LDL | 2.127（1.773-2.552） | ＜0.001 |
| ALT | 1.032（1.026-1.038） | ＜0.001 |
| Cr | 1.022（1.006-1.038） | 0.006 |
| TyG*10 | 1.172（1.145-1.200） | ＜0.001 |
| TyG-BMI*0.1 | 1.304（1.262-1.349） | ＜0.001 |

Supplemental Table 2: Univariate Logistic Regression of HUA
